# Integrating electronic healthcare records of armed forces personnel: Developing a framework for evaluating health outcomes in England, Scotland and Wales

**DOI:** 10.1016/j.ijmedinf.2018.02.012

**Published:** 2018-05

**Authors:** Daniel Leightley, Zoe Chui, Margaret Jones, Sabine Landau, Paul McCrone, Richard D. Hayes, Simon Wessely, Nicola T. Fear, Laura Goodwin

**Affiliations:** a King’s Centre for Military Health Research, Institute of Psychiatry, Psychology & Neuroscience, King’s College London, United Kingdom; b Biostatistics & Health Informatics, Institute of Psychiatry, Psychology & Neuroscience, King’s College London, United Kingdom; c Health Services & Population Research, Institute of Psychiatry, Psychology & Neuroscience, King’s College London, United Kingdom; d Psychological Medicine, Institute of Psychiatry, Psychology & Neuroscience, King’s College London, United Kingdom; e Academic Department of Military Mental Health, Institute of Psychiatry, Psychology & Neuroscience, King’s College London, United Kingdom; f Department of Psychological Sciences, University of Liverpool, United Kingdom

**Keywords:** A&E, accident and emergency, APC, admitted patient care, CHI, community health index, DOB, date of birth, EHRs, electronic healthcare records, HES, health episode statistics, ISD, information services division, ICD-10, international classification of diseases, 10th revision, KCMHR, King’s College Centre for Military Health Research, NHS, National Health Service, OPCS-4, OPCS classification of interventions and procedures version 4, SAIL, secure anonymised information linkage, UK, United Kingdom, Hospital episode statistics, Electronic health records, Hospital admission, Secondary care, Big data, Data linkage

## Abstract

**Background:**

Electronic Healthcare Records (EHRs) are created to capture summaries of care and contact made to healthcare services. EHRs offer a means to analyse admissions to hospitals for epidemiological research. In the United Kingdom (UK), England, Scotland and Wales maintain separate data stores, which are administered and managed exclusively by devolved Government. This independence results in harmonisation challenges, not least lack of uniformity, making it difficult to evaluate care, diagnoses and treatment across the UK. To overcome this lack of uniformity, it is important to develop methods to integrate EHRs to provide a multi-nation dataset of health.

**Objective:**

To develop and describe a method which integrates the EHRs of Armed Forces personnel in England, Scotland and Wales based on variable commonality to produce a multi-nation dataset of secondary health care.

**Methods:**

An Armed Forces cohort was used to extract and integrate three EHR datasets, using commonality as the linkage point. This was achieved by evaluating and combining variables which shared the same characteristics. EHRs representing Accident and Emergency (A&E), Admitted Patient Care (APC) and Outpatient care were combined to create a patient-level history spanning three nations. Patient-level EHRs were examined to ascertain admission differences, common diagnoses and record completeness.

**Results:**

A total of 6,336 Armed Forces personnel were matched, of which 5,460 personnel had 7,510 A&E visits, 9,316 APC episodes and 45,005 Outpatient appointments. We observed full completeness for diagnoses in APC, whereas Outpatient admissions were sparsely coded; with 88% of diagnoses coded as “*Unknown/unspecified cause of morbidity*”. In addition, A&E records were sporadically coded; we found five coding systems for identifying reason for admission.

**Conclusion:**

At present, EHRs are designed to monitor the cost of treatment, enable administrative oversight, and are not *currently* suited to epidemiological research. However, only small changes may be needed to take advantage of what should be a highly cost-effective means of delivering important research for the benefit of the NHS.

## Highlights

• A framework which integration national Electronic Healthcare Record datasets from England, Scotland and Wales is proposed.
• Variable similarity is used to develop a schema which allows for variables to be linked and combined across the nations.
• Evaluation of integration shows that it is possibly to perform data linkage across the nations.

## 1 Background

Routinely collected Elxectronic Healthcare Records (EHRs) can be used to evaluate disease prevalence, tendencies, and to perform epidemiological analyses [1]; investigate quality of care and to improve clinical decision-making, which can influence patient outcome and care [[2], [3]]. In recent years, there has been a growth in the use of EHRs in the field of Big Data analytics, which is the analysis and evaluation of large datasets, to answer specific research questions [[4], [5]]. The term “Big Data” [6] has been used in a number of health settings, it is synonymous with the meaningful analyses of EHRs to identify health movements and associations [7]. Further, Schneeweiss et al. [8] summarises the potential applications into two key areas: generation of knowledge to improve the effectiveness of treatment; and to predict the outcome of treatment and diagnoses.

Globally, the development and use of EHRs is increasing, alongside Big Data innovation in a number of fields [[8], [9], [10]]. Standardised EHR systems are difficult to implement in larger countries, those which have complex political structures or multiple private entities [11], such as the United States. In the United Kingdom (UK), health and social care is devolved to national Government and local agencies using propriety systems which are not interconnected or able to identify patients who relocate from a nations [[11], [12], [13]]. In the UK, it is estimated that 53,000 individuals migrate from England and Wales to Northern Ireland and Scotland; with 46,800 individuals migrating from Northern Ireland and Scotland to England and Wales each year [14]. At present, migration statistics are not reported separately, making it difficult to determine cross-border migration. EHRs which represent the same patient across the three nations show great promise and could be used to identify risk, inform healthcare policy, service provision and improve health and social outcomes [[13], [15], [16]]. Using a cross-border cohort might provide the foundation of creating such a system. While several studies have sought to create national datasets of health and social care [[4], [17]], to our knowledge, no studies exist which integrate EHRs from multiple nations into a single repository for research.

There are multiple challenges in integrating EHRs in the UK. Firstly, there is no system or framework which uniquely identifies a person (*e.g.* unique ID number for each citizen) across public services such as welfare, housing, education and health [18]. Those registered with the National Health Service (NHS) of England and Wales are assigned a unique 10-digit number, which is used as the sole identifier in healthcare [19]. Similarly, in Scotland a person is assigned a Community Health Index (CHI) number which is used in the same manner. Second, EHRs provided by England [20], Wales [[4], [17]] and Scotland [21] are distinctive in structure, collection and management. Third, EHR data from each of these data sources include various data types, from structured information such as drug prescriptions, diagnoses and treatment, to unstructured data such as clinical notes and patient self-reported illness [11]. Finally, there is a dearth of literature which describes how to undertake EHR data linkage from multiple data sources [22].

Current research efforts have been directed towards modelling and predicting single conditions (*e.g.* depression, diabetes and epilepsy) and outcomes [[3], [16], [23], [24]]. In this work, we study a UK Armed Forces cohort [[25], [26]] (which includes individuals from England, Scotland and Wales) that has been linked separately to three sources of national secondary healthcare data. These data have then been integrated to form a patient-level dataset across 3 nations which include A&E, Outpatient Care and Admitted Patient Care (APC). The subsequent dataset is unique, population-based longitudinal dataset that contains a wide range of physical and mental health indicators of serving and ex-serving UK Armed Forces personnel. The objectives of this work are; 1) to develop and describe a methodology which integrates EHRs of Armed Forces personnel in England, Scotland and Wales 2) examine healthcare utilisation within these data and 3) evaluate characteristics of admission, record completion and diagnoses.

## 2 Methods

**Fig. 1:**
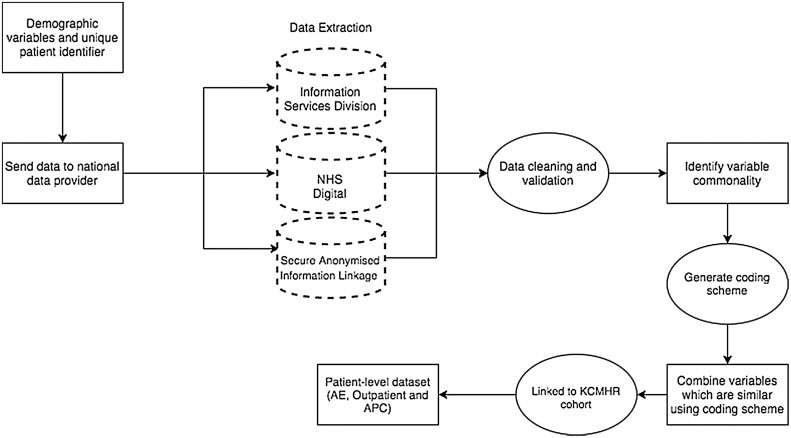
Overview of the data linkage process and formation of patient-level dataset.

The proposed framework is illustrated in Fig. 1, and is represented by the following stages:

1. Demographic identifiers of Armed Forces personnel are sent to each data provider;
2. EHRs are extracted and sent to the research team by data providers;
3. EHRs are cleaned and validated;
4. Variables are linked using commonality;
5. EHRs for each patient are integrated to generate a patient-level dataset.

### 2.1 King’s centre for military health research (KCMHR) cohort

UK Armed Forces personnel receive all secondary care from the NHS while in service in the UK and after they have left service from the NHS. This study uses the example of an Armed Forces population to integrate data from England, Scotland and Wales. The reasons for this are 1) they frequently move/relocate, so it is important to capture EHRs from across the nations and 2) they may have different health needs due to being exposed to a high level of physical and mental strain. This study used the KCMHR cohort, which is a longitudinal cohort of the UK Armed Forces (full details of the methods and sample description were previously reported in [[25], [26]]), to develop methodologies to integrate EHR data. Briefly, data was collected in 2004–2006 from a sample of personnel deployed to the Iraq war during 2003 and from a sample of personnel serving in the UK Armed Forces but who did not deploy at that time (phase 1). Phase 1 participants were then followed up in 2007–2009, where two new samples were introduced to represent the demographics of the Armed Forces at that time point (phase 2). Individuals who took part in phase 1 only were not included in the study as they had not been asked for consent to access their medical records. Participants were only included in the study if they provided content for us to access their medical records, but the authors also sought and obtained a section 251 of the National Health Service Act 2006 [27] approval to allow transfer of confidential participant information to data providers for processing (to facilitate data linkage). In total, 86.11% of phase 2 respondents provided informed consent to access their healthcare records, resulting in a final cohort of 8,602. The consenting cohort available for data extraction and integration comprised 7,661 (89.06%) males and 941 (10.94%) females, which is in line with the demographics of the UK Armed Forces.

### 2.2 Data providers

EHRs are managed by NHS Digital in England [20], Secure Anonymised Information Linkage (SAIL) in Wales [[4], [17]] and Information Service Division (ISD) in Scotland [21]. Table 1 illustrates the datasets, periods of interest and requested number of variables. Variables that were comparable (*e.g.* similar in content and type) between data providers were requested. Where available, data was requested for the financial years 2003/04 to 2013/14 or closest available.

**Table 1:** Defines the terminology used by data providers, data periods of interest and number of variables requested.

Terminology		NHS Digital	Secure Anonymised Information Linkage (SAIL)	Information Services Division (ISD)
Accident and Emergency (A&E)	Year range	2007/08–2013/14	2009/10–2013/14	2003/04–2013/14
	Dataset	Accident and Emergency	Emergency Department Data Set	Accident and Emergency
	Variable Count	142	69	42
Admitted Patient Care (APC)	Year range	2003/04–2013/14	2003/04–2013/14	2003/04–2013/14
	Dataset	Admitted Patient Care	Patient Episode Database for Wales	Scottish Morbidity Records 01
	Variable Count	265	115	36
Outpatient	Year Range	2003/04–2013/14	2004/05–2013/14	2003/04–2013/14
	Dataset	Outpatient	Outpatient	Outpatient
	Variable Count	96	46	32

### 2.3 Extracting electronic healthcare records

Data extraction was performed independently of the authors through the data providers to preserve privacy. Providers were supplied a set of demographic variables; 10-digit NHS number (where available), first name, middle name, surname, gender and date of birth (DOB). When providing extracted data, providers removed all demographic identifiers (*e.g.* first name, last name); to enable identification the authors supplied a unique identifier to represent each participant. For clarity, the extraction process is described hereafter, data extraction had **no impact** on the creation of the framework or integrated datasets.

NHS Digital required a valid NHS number to identify any individual and extract Health Episode Statistics (HES) EHR data. Valid NHS numbers were mapped with an internal identifier, the HES ID. EHRs were extracted if any of the following conditions were met: (1) match on NHS number, gender and DOB; (2) match on NHS number, partial match for gender or DOB; (3) match on NHS number only.

SAIL operated a two-stage extraction process to preserve privacy. First, demographic variables were supplied by the Data Provider to NHS Wales Informatics Service [28] where they were cross-referenced using a fuzzy matching procedure to create the Anonymised Linkage Field (a unique SAIL databank identifier). Second, NHS Wales Informatics Service supplied the Anonymised Linkage Field to the SAIL databank to join with record details provided to SAIL to produce an anonymised version of each dataset, linkable at the individual level to EHR datasets within SAIL.

ISD did not require a NHS/CHI number for matching. Participant demographics were cross-matched using probabilistic linking to identify a) valid NHS/CHI number; b) to confirm the NHS/CHI number is valid. EHRs which belonged to a valid CHI number were identified and extracted.

Data from each provider was supplied in financial year using a comma separate file format.

### 2.4 Developing the integrated dataset using variable commonality

The datasets contain many variables (Table 1); however, it is not practical to directly link, integrate, or combine variables which may be named or categorised differently. To overcome this, we assessed variable commonality; defined as those variables which represent similar data mediums but categorised in a different manner. The framework solely categorised variables based on the data they hold. For example, each dataset contained a variable which identified speciality; however, the coding schemes were different, therefore a new coding scheme was developed to be usable across all data providers. Table 2 defines the variable categories of interest. Variables which did not match the definition, or were only available for one data source were not selected unless they contributed and provided valuable insight (*e.g.* method of admission). Discussions were held within the research team to discuss variable categorisation, matching and development of coding schemes.

**Table 2:** Stipulates the definition used for associating variables to a commonality category.

Category	Criteria
Admittance/Discharge	Variables that provide information on the admission and discharge of a patient. This includes admission and discharge date, episode information, time of admission, source of admission and destination of patient upon discharge.
Diagnosis/Classification	Variables that provide information on the diagnosis of the patient, including ICD-10 coding and date of diagnosis, A&E coding or local diagnoses coding system.
Treatment/Procedure/Investigation	Variables that provide information on the treatment/procedures undertaken, including OPCS Classification of Interventions and Procedures version 4 coding, or local diagnoses coding system.
Care Provider	Variables that provide information on the provider of care, including geographical location and provider type.
Care Speciality	Variables that provide information on the speciality of care, including consultant association, department of care and clinical staff role.
Costing/Resources	Variables that provide information on the cost of care, including cost of treatment, staffing costs and direct costs incurred by the care provider.

NHS Digital (HES datasets), the largest data provider of EHRs in the UK, was selected as the anchor for comparison to other datasets.

Variable commonality was undertaken in three stages. First, **individually** for each data provider; A&E, APC and Outpatient variables were grouped using the categories defined in Table 2. Second, variables that matched across the datasets were identified (*e.g.* admission date, discharge date, diagnoses and treatment). Finally, variables that were similar in nature were identified and a common coding scheme was developed to reflect coding schemes used by data providers (*e.g.* source of admission, source of discharge and source of injury) with the objective of having a single coding scheme. Supplement 1 outlines variables linked for each dataset and data provider.

Admitted Patient Care EHRs across the data providers, include details of the patient, when they were treated, where they were treated and what they were treated for. As illustrated in Table 1, APC records contain the largest number of variables compared to other departments. Variables identified include admission/discharge date, care provider, speciality and costing codes. For variables such as consultant speciality and admission/discharge source a national coding scheme was used. We developed a common coding scheme which was inclusive and representative of national coding schemes. Codes which were ambiguous or not an identical match to other nations were retained. Primarily, diagnoses were coded using the International Classification of Diseases, 10th revision (ICD-10) and interventions and procedures are coded using OPCS Classification of Interventions and Procedures version 3 or 4 (OPCS-4).

Outpatient appointments were recorded across the data providers, including appointment date, appointment treatment, care provider and outcomes. Variables differed widely across the data providers due to service provision and method of recording the visit. Variables identified for joining included appointment date, status of appointment attendance, diagnosis and speciality. For variables such as attendance type and appointment source, a common coding scheme was developed bringing together individual nation coding schemes. Codes which were ambiguous (without a suitable definition) or not an identical match were retained separately. Diagnoses, interventions and procedures were coded using ICD-10 and OPCS across the data providers.

Accident and Emergency attendance was recorded differently across the data providers, and for NHS Digital HES data were different between hospital trusts. Variables such as admission date, treatment time, conclusion time and discharge time/location were linked based on commonality. Major differences were observed when coding the reason for the visit to A&E; unlike APC, which utilises the ICD-10 coding system across the combined dataset, A&E does not use a national standard. For example, within England, care providers utilise a local coding system, ICD-10 coding or a NHS England coding standard [[29], [30]].

### 2.5 Integrated patient record and data cleaning

A patient-level EHR dataset was constructed using a five-stage integration process. First, Continuous Inpatient spells were computed for APC in HES, ISD and SAIL separately. A Continuous Inpatient spell is considered as a continuous period of care within the NHS, regardless of any transfers between care providers that may take place [31]; it can comprise one or more providers but starts when a decision has been made to admit the patient (admission date), and ends when the patient dies or is discharged from hospital (discharge date). APC records are treated differently to other EHRs (*e.g.* A&E, Outpatient) as a patient can have multiple episodes of care relating to a single admission into hospital. Second, admission and discharge date (A&E, Outpatient and APC) were checked to ensure consistency and that a valid date was present. Third, duplicate records (A&E, Outpatient and APC) relating to the same period of care or admission were identified and excluded. Fourth, EHR (A&E, Outpatient and APC) for the same individual were brought together within HES, SAIL and ISD to create a personal admission history for England, Wales and Scotland respectively using the unique identifier. Finally, admission history across the nations was merged to the KCMHR Armed Forces cohort to create a patient-level history for A&E, APC and Outpatient care.

### 2.6 Data analysis

This work compared the matched sample (n = 6,336) with the non-matched sample (n = 2,266) to assess differences on a range of demographic factors. Analyses including median and frequencies were computed in Stata 12 [32]. In addition, the following descriptive analyses were performed:

• **Linkage rate** overall and by nation were computed using record matching information supplied by national data providers. The analyses were undertaken using those who gave consent for their medical records to be accessed (n = 8,602). Results were presented as frequency and percentage values. Students’ *t*-test was performed to identify significance in the matching.
• Departmental utilisation was calculated based on the number of visits a patient made to the department. Gender frequencies and percentages were computed to provide a breakdown of departmental admissions.
• **Common conditions** were calculated by summing the number of events for each 3-digit ICD-10 code and represented as a frequency. Gender frequencies and percentages were computed for each common condition.
• The total numbers of hospital visits were calculated by summing the number of events per participant matched for each department. The frequency, median and inter-quartile range (IQR) were computed at the patient-level as the data was not normally distributed.
• Variable completeness was calculated as the number of valid variable entries compared to missing or null entries (where 0 is treated as a valid entry). Valid variable entries were represented by records which have a value which is not null or missing. Results were presented as percentages.

It should be noted that the unit of analysis is the patient, rather than hospital, geographical region or nation.

## 3 Results

### 3.1 Overview of the data linkage

**Fig. 2:**
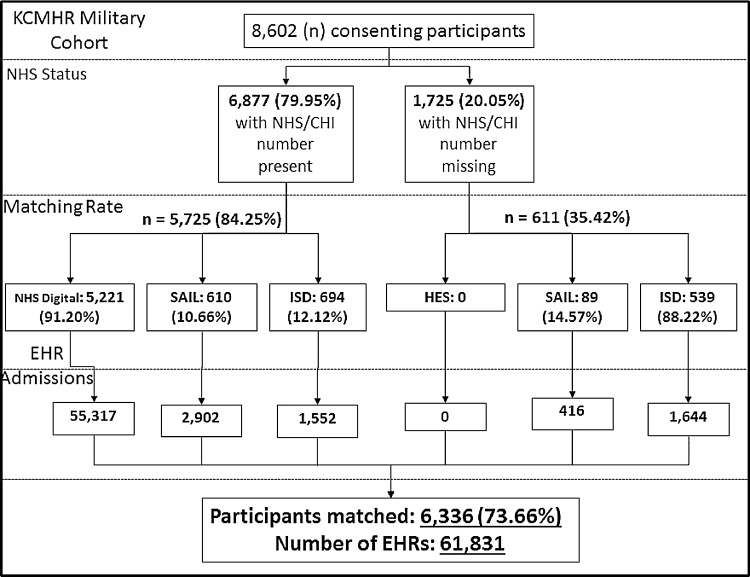
Data extraction, number of participants matched and the total number of EHR for each data source divided by those with and without an NHS/CHI number. Note: A participant can appear in multiple nation data providers. Percentage figures are cascading, where the percentage is out of the preceding value.

The completeness of demographic variables, which may impact data extraction, contained within the KCMHR Armed Forces cohort is presented in Table 3. The number of participants matched was 6,336 (74%) as illustrated in Fig. 2. The proportion matched was greater in those with a NHS/CHI number compared to those without, with the latter restricted to matching in Wales and Scotland. This was due to the NHS Digital in England requiring a valid NHS number for matching (blocking variable). Of individuals matched, 4,460 were matched in England only (71%), 257 were matched in Wales only (4%), 826 in Scotland only (13%) and 793 were matched in more than one nation (12%). 5,460 participants had a hospital contact during the period of interest.

**Table 3:** Completeness of KCMHR demographics (n = 8,602). ^1^NHS/CHI number is complete if length is greater than 8. CHI number serves the same purpose as NHS number for Scotland. ^2^Valid if length 1 or greater. ^3^Valid if date of birth is present e.g. DD/MM/YY or DD/MM/YYYY.% represents row percentages.

	NHS Number/CHI Number^1^	Initial	Forename^2^	Surname^2^	Gender	Date of Birth^3^
Variable Completeness	6877 (79.95%)	8179 (95.08%)	8413 (97.8%)	8602 (100%)	8602 (100%)	8597 (99.94%)
Missing Values	1725 (20.05%)	423 (4.92%)	189 (2.2%)	0	0	5 (0.06%)

### 3.2 Overview of healthcare utilisation

The highest proportion of hospital visits recorded for England was 5,221 participants compared to Scotland with 1,233 participants or Wales with 699 participants (Fig. 2). The overall number of hospital episodes for each nation and department is presented in Table 4. In summary, A&E had a median of 2 (interquartile range [IQR] 2) admissions per participant, APC had a median of 2 (IQR: 2) episodes per participant and Outpatient had a median of 5 (IQR: 8) appointments per participant.

**Table 4:** Represents the number of episodes and participant numbers for each department and nation. Percent values represent the percentage of the matched sample (n = 6336).

Department		NHS Digital	Secure Anonymised Information Linkage	Information Services Division
Accident and Emergency	Episodes	6775	392	343
	Participant	2873 (45.44%)	163 (2.77%)	206 (3.25%)
Admitted Patient Care	Episodes	7516	444	577
	Participant	2970 (46.77%)	176 (19.3%)	251 (3.96%)
Outpatient	Episodes	41,026	1703	2276
	Participant	4300 (67.87%)	240 (3.79%)	435 (6.87%)

### 3.3 Healthcare utilisation and diagnoses in accident and emergency

A total of 3,192 participants had 7,510 admissions to A&E (2,813 (88.13%) males and 379 (11.87%) females). 253 variables were available for linkage, we derived 102 common variables. Out of the admissions to A&E, 90.32% were discharged or transferred to a ward on the day of admission, with 9.62% spending at least 24 h within A&E. There were five methods identified for recording reason for an admission; for England: ICD-10, local coding and regional coding schemes, Wales: ICD-10 and regional coding schemes, finally Scotland: ICD-10 and free-text input. The most common mode of attendance was self-presentation (75.50%) followed-by road ambulance (7.49%) and then hospital transfer (6.52%).

Variable completeness of the original data providers (Table 1), measured as a valid value being present was 47.12% (Table 5). This reflects a code presence of less than 60% for A&E admissions with Reason for Admission, Treatment and Investigation. It is important to note that Reason for Admission relates to the presenting complaint(s) of the patient upon admission, in England up to 12 can be recorded. As illustrated in Table 5, completeness for Reason for Admission was 56.43%, meaning that almost half of all admissions did not have any presenting complaint or assigned diagnoses. Conversely, variables related to the patient admission, care provider and cost were consistently coded (>90%).

**Table 5:** Variable completeness for a sample of common variables formed in England, Scotland and Wales.

Data Source	Common Variable (national assigned variable name)	NHS Digital	Information Services Division	Secure Anonymised Information Linkage
Accident & Emergency	Reason for Admission^1^	42.44%	67.93%	58.93%
	Attendance Category (attendance_cat)	100%	100%	100%
	Admission Time (time_arrival)	87.13%	93.76%	90.51%
	Arrival Mode (arrival_mode)	100%	97.96%	100%
	Provider Code (provider_code)	100%	100%	100%
Admitted Patient Care	Primary (1st) Diagnosis (diag_01)	100%	100%	100%
	Admission Source (admin_source)	98.59%	100%	100%
	Main Speciality (main_speciality)	100%	97.13%	98.08
	Operation (oper_01)	100%	72.62%	68.52%
	Discharge Method (dist_meth)	100%	77.34%	79.09%
Outpatient	Attend (attended)	100%	77.07%	80.28%
	Attended Type (attend_type)	100%	100%	100%
	Main Speciality (main_ speciality)	100%	100%	100%
	Referral Source (ref_source)	100%	78.60%	100%
	Diagnosis (diag_01)^2^	97.98%	100%	100%

1 Represents a group of variables which describe the anatomical area, side body and presenting diagnosis.

2 “*Unknown and unspecified causes of morbidity*” assigned to 87.57% of all appointments.

### 3.4 Healthcare utilisation and diagnoses in admitted patient care

A total of 3,324 participants had 9,316 episodes, 2,842 were male (85.50%) and 482 were female (14.50%); 416 variables were present from the nations, with 89 common variables identified to represent a single episode. Overall, variable completeness across the derived dataset, in terms of a value being present was 78.06% (Table 5). All episodes were recorded with at least one diagnosis using ICD-10 coding scheme with full completeness across all three nations. There was high variable completeness amongst Admittance, Diagnosis/Classification and Care Speciality variable groups (>85%). Conversely, variables related to the patient discharge, episode order and cost were more likely to be missing data (<80%).

Unlike A&E or Outpatient EHR, each record was accompanied by a diagnosis; completeness was 100%. In this population, 17,541 individual ICD-10 diagnoses, representing 963 ICD-10 (3-digit) disease groups including primary and secondary diagnoses were recorded. Aggregating the ICD-10 code *Z51* representing “*Other medical care*” was the most common (Table 6). Predominately, males across all codes are more likely to be admitted than females (except in the event of “*Outcome of Delivery*” which relates to child birth); this is not unexpected, as the cohort is largely male. The most common source of an admission was the residential home of the patient (90.07%) and other NHS hospitals was the second most common source (8.08%). Five participants died during a hospital episode.

**Table 6:** Most common ICD-10 codes assigned during Admitted Patient Care visit with gender comparison.

	ICD-10 Code	Description	Occurrence (n = participants)	Male (n = 874)	Female (n = 109)
1	Z51	Other medical care	490 (n = 116)	99 (85.34%)	17 (14.66%)
2	Z86	Personal history of certain other diseases	336 (n = 190)	159 (83.68%)	31 (16.32%)
3	I10	Essential (primary) hypertension	295 (n = 151)	140 (92.72%)	11 (7.28%)
4	Z37	Outcome of delivery	281 (n = 198)	0	198 (100%)
5	Z30	Contraceptive management	272 (n = 262)	247 (94.27%)	15 (5.73%)
6	R10	Abdominal and pelvic pain	266 (n = 221)	155 (70.14%)	66 (29.86%)
7	M23	Internal derangement of knee	265 (n = 217)	208 (95.85%)	9 (4.15%)
8	F17	Mental and behavioural disorders due to use of tobacco	259 (n = 190)	168 (88.42%)	22 (11.58%)
9	Z72	Problems related to lifestyle	253 (n = 197)	183 (92.89%)	14 (7.11%)
10	M54	Dorsalgia	222 (n = 123)	102 (82.93%)	21 (17.07%)

### 3.5 Healthcare utilisation and diagnoses in outpatient services

A total of 4,810 participants had 45,005 appointments, with 4,178 (86.86%) male and 632 (13.14%) female. Multiple coding systems were employed to encode diagnoses of an outpatient visit, including ICD-10 and free text. The most common ICD-10 code is R69, defined as “*Unknown and unspecified causes of morbidity*” assigned to 87.57% of all appointments. Only 5.16% of appointments were not coded with any type of diagnosis. England had the largest number of visits with a proportion of 91.34% compared to Scotland 4.72% and Wales 3.94%.

In total, 174 variables, which represent all three nations, were grouped resulting in 46 common variables being defined. Overall completeness of the derived variables, in terms of a value being present was an average of 57.11% (Table 5). Appointments were coded with an appointment date, with the reason for attendance fully coded. Conversely, there was poor coding for appointment waiting time, priority status, diagnoses and speciality (<70%).

## 4 Discussion

Our work introduces a framework to integrate data from national providers to produce a combined dataset of secondary health for serving and ex-serving Armed Forces personnel. This work can also be applied to other settings and populations. We have shown that a linked dataset containing national EHRs can be created and used to evaluate healthcare utilisation across England, Scotland and Wales. However, it is important to acknowledge that we do not know how many persons with an admission were not detected due to the matching and extraction methodology employed by data providers. Using linked EHRs, clinicians and researchers can monitor and improve admissions, quality of care, improve clinical decision making, and to influence patient outcomes and care. We have been able to use the data to show the frequency of APC, Outpatient and A&E visits, diagnosis and arrival mode for Armed Forces personnel. Conversely, we have identified disparities between data providers and their methods of recording, validating and storing of data which has been shown to impact data quality and reliability. Similarly, data completeness and accuracy in assigning presentation or diagnoses code values are of concern in A&E and Outpatient data across the nations. For Outpatient EHRs, diagnoses and main procedure variables were not a mandatory requirement, however recording is increasing over time [33]. Nevertheless, APC has been shown to consist of valid data points including diagnoses, treatments and operations, and admission/discharge dates. To our knowledge, this is the first to both link secondary care datasets across England, Scotland and Wales and to analyse Armed Forces personnel EHR data.

The ability to obtain EHR from multiple national providers and integrate to an existing cohort allows greater insight into patient admission patterns and history [[2], [34], [35]]. However, EHRs alone do not provide sufficient context to enable detailed evaluation of the data and therefore additional data providers and cohorts (e.g. National Joint Registry [36], Cancer Registry [37]) are used to provide personal identifiers. The Hertfordshire Cohort Study [3] linked hospital admissions to an existing cohort, the study was limited to a single English county (Hertfordshire county). The authors had difficulty obtaining EHRs for the cohort, similar to our own difficulties with national data providers performing data linkage using different algorithms. In Scotland, the Scottish Health Surveys Cohort [5] is an example of a data linkage between a cohort and health and social care in secondary care. The study linked Outpatient, APC, Mental Health and Outpatient records at the patient-level to provide detailed healthcare patterns.

### 4.1 Limitations and challenges for sustainability

For our methodology to be a functional resource it is important to be aware of the limits of health data and integration. Personal identifiers were obtained via the KCMHR cohort study, with permission from participants, and included participants’ first name, last name, date of birth gender, and National Health Number which can be difficult to obtain and record correctly. NHS number was not available for 20.05% of participants. The matching process undertaken by NHS Digital set NHS number as a mandatory variable, resulting in a lower success rate. However, ISD in Scotland and SAIL in Wales did not place this same requirement. We did not request data from Northern Ireland. This was because of the complex structures surrounding EHRs in Northern Ireland. No central organisation is responsible, instead it is delegated to the local trust. In addition, there are security concerns surrounding former Armed Forces personnel.

There are several problems with using national EHR datasets such as HES, ISD or SAIL when integrating these datasets. The complexity of gathering data at data centres and the large number of organisations and institutions which can provide submissions can give rise to reduced accuracy of the EHR including diagnoses, outcomes and patient management [[38], [39]]. Further, completeness of records is poor, particularly in Outpatient and A&E EHRs, giving rise to difficulty in linking variables and making any statistical analyses troublesome [[3], [5], [24]].

We were limited to the variables requested and provided by national data providers, to enable linkage. We undertook a pragmatic approach to matching variables based on commonality using variable definitions provided by the data providers. This is a time-consuming exercise, and might prove an obstacle in even larger datasets. Further, national coding schemes, may have been interpreted differently by hospital trust and coder leading to additional confusion and coding bias. It is important to be aware that though EHRs are widely used in research, offering a broad range of information about treatment, diagnoses and care, there are issues relating to determining data quality, completeness of data and drawing conclusions from the data [[40], [41], [42]].

### 4.2 Unanswered questions and future developments

Our initial examination of the dataset has proved useful in identifying the most common diagnoses, types of A&E admissions and number of Outpatient appointments for Armed Forces personnel. Further analysis of the dataset is required to evaluate clinical practices, common diagnoses stratified by patient characteristics and variable coding accuracy to improve dataset recording and retention. This could include assessing A&E trends in admission, prevalence of A&E admissions resulting in an APC episode and waiting times. Alternately, further data linkage to primary healthcare records might make it possible to identify pre and post follow-up health of individuals who have had a contact with secondary care. This could improve the quality of analysis and enable refinement of queries used on the dataset to improve the quality of the dataset.

In the future, there is potential to develop a more responsive, real-time and useful linked dataset based on the framework and methodologies presented in this work. This would require more regular and frequent updates from data providers, including more efficient data linkage of cohort users.

## 5 Conclusions

It was possible to integrate secondary health records to create a combined dataset of attendances to NHS hospitals in England, Scotland and Wales. This is the first to combine and present analyses of EHRs from Armed Forces personnel in the UK (excluding Northern Ireland). The dataset was developed using variable commonality and included both serving and ex-serving Armed Forces personnel, enabling evaluation of healthcare utilisation. The NHS of England, Scotland and Wales obtain, store and process EHRs using different methodologies. Scotland and Wales utilise participant demographics without the need of a NHS number whereas matching in England requires an NHS number. Future work should seek to harmonise the protocols for national linkage and seek a uniform UK-wide policy on EHRs for research. The increasing shift towards the use of EHR by health trusts presents an opportunity to monitor admissions, diagnoses and outcomes to inform public health policy and service provision.

## Ethical approval and consent to participate

Ethical approval was obtained from the London-Dulwich NHS Research Ethics Committee in November 2014 (REC no: 07/Q0703/36). Further, a Section 251 of the National Health Service Act 2006 (Ref no: 15/CAG/0136) was obtained.

## Consent for publication

None required.

## Availability of data and materials

Coding schemes and syntax used to perform data linkage are available upon request from the corresponding author.

## Fundings

This work is funded by the Economic and Social Research Council (grant number ES/L014521/1), DL and ZC were funded by the grant. Over the course of this work RDH was funded by a Medical Research Council Population Health Scientist Fellowship (grant number MR/J01219X/1). RDH and SL have received salary support from the National Institute for Health Research Mental Health Biomedical Research Centre at South London and Maudsley NHS Foundation Trust and King’s College London. The views expressed are those of the author(s) and not necessarily those of the NHS, the NIHR or the Department of Health.

## Conflict of interest

RDH has received research funding from Roche, Pfizer, Janssen, Lundbeck and In-Silico-Bioscience. SW is advisor to the British Army in psychiatry and a trustee of Combat Stress. This work was undertaken prior to NTF’s appointment to the Independent Group Advising on the Release of Data. NTF is a trustee of The Warrior Programme. DL, ZC, SL, PM, MJ and LG declare that they have no competing interests.

## Author contributions

DL conducted the data linkage, data analysis and wrote all drafts of the manuscript. ZC provided advice on the data linkage, commented on drafts of the paper. MJ has provided guidance during the work and commented on drafts of the paper. SL, PM, RDH, SW and NTF participated in design of the work and commented on drafts of the paper. LG designed the work, sought and obtained funding, oversaw data collection, analysis, linkage and commented on all drafts of the paper. All authors have read and approved the final manuscript.

**Summary table**

*What was already known?*

• Electronic Healthcare Records are used to capture summaries of care and contact to healthcare services; in the United Kingdom they are intended to financially reimburse hospital trusts for care provided.
• In the United Kingdom; England, Scotland and Wales maintain separate data stores for Electronic Healthcare Records with responsibility being devolved to National Governments. This has introduced a lack of uniformity, making it difficult to evaluate care, diagnosis and treatment across the entire United Kingdom.
• Propriety systems are used by England, Scotland and Wales National Health Services which makes it challenging to perform analyses across England, Scotland and Wales.

*What has this study added to the body of knowledge?*

• This paper presents the first framework to integrate national Electronic Health Records. It has been developed with collaboration from health professional and could be rolled out nationally to assist in United Kingdom wide research.
• This paper found that variable completeness across the nations was varied, with Outpatient care being sparsely coded making it challenging for use in epidemiological research.
• This paper highlights the types of analyses which can be performed when undertaking United Kingdom wide linkage, with the potential of combing additional data modalities.
