# Supplementary material for: Integrating electronic healthcare records of armed forces personnel: Developing a framework for evaluating health outcomes in England, Scotland and Wales
Source: Int J Med Inform. 2018 May;113:17–25. doi: 10.1016/j.ijmedinf.2018.02.012 (PMC5887874; doi:10.1016/j.ijmedinf.2018.02.012)
Supplement: Supplementary file 1 [file mmc1.docx]

**Supplements**

**Supplement 1 – Variable Framework**

**Admitted Patient Care**

| **Category** | **Common Variable Name** | **England – Variable** | **Scotland – Variable** | **Wales – Variable** |
| --- | --- | --- | --- | --- |
| Admittance  Discharge | admin_date^1^ | admidate | admission_date | admis_dt |
|  | admin_meth^2^ | admimeth |  | admis_mthd_cd |
|  | admin_source^2^ | admisorc | admission_transfer_from | admis_source_cd |
|  | dis_date^1^ | disdate | discharge_date | disch_dt |
|  | dis_dest^2^ | disdest | discharge_transfer_to | disch_destination_cd |
|  | dis_meth^2^ | dismeth | discharge_type | disch_mthd_cd |
|  | epi_start^1^ | epistart |  | epi_str_dt |
|  | epi_end^1^ | epiend |  | epi_end_dt |
|  | epi_order^1^ | epiorder |  | epi_num |
|  | epi_type^2^ | epitype |  |  |
|  | patient_type | classpat | admission_type | pat_class_cd |
|  | ready_date | disreadydate | ready_for_discharge_date |  |
| Diagnosis  Classification | diag_01-diag_20^1^ | diag_01-diag_20 | main_condition  other_condition_1-other_condition_5 | diag-diag_n |
|  | cause^1^ | cause |  |  |
| Treatment  Procedure  Investigation | oper_01-oper_23^1^ | opertn_01-opertn_24 | main_operation  other_operation_1-other_operation_3 | oper_cd_123 |
|  | oper_date_01-oper_date_24^1^ | opdate_01-opdate_24 | date_of_main_operation |  |
| Care Speciality | main_speciality^2^ | mainspef | specialty | con_spec_main_cd |
|  | treat_speciality^2^ | tretspef |  | con_spec_cd_of_treat |
| Costing  Resources | dominant_procedure^1^ | domproc |  |  |
|  | hrg_nhs^1^ | hrgnhs |  | hrg_localpayment_cd |
|  | hrg_nhs_svn^1^ | hrgnhsvn |  | hrg_referencecost_cd |
|  | pur_val^1^ | purval |  |  |
| Care Provider | site_treatment^1^ | sitetret |  | prov_site_cd |
|  | provider_code^1^ | procode | provider_code |  |
|  | provider_type^1^ | protype |  | curr_prov_unit_cd |

^1^Variable retained without coding scheme. ^2^Variabe retained with a coding scheme.

**Accident and Emergency**

| **Category** | **Common Variable Name** | **England – Variable** | **Scotland – Variable** | **Wales – Variable** |
| --- | --- | --- | --- | --- |
| admittance  discharge | arrival_mode^2^ | aearrivalmode | arrivalmodecode | arrival_mode |
|  | attendance_cat^2^ | aeattendcat | attendancecategorycode | attend_category |
|  | discharge_dest^2^ | aeattenddisp | dischargedestinationcode |  |
|  | incident_location^2^ | aeincloctype | placeofincidentcode |  |
|  | activity_injured^2^ |  | activitywheninjuredcode | activity |
|  | intent_of_injury^2^ |  | intentofinjurycode | attend_group |
|  | patient_group^1^ | aepatgroup |  |  |
|  | referral_source^2^ | aerefsource | referralsourcecode |  |
|  | date_arrival^1^ | arrivaldate | arrivaldate | admin_arr_dt |
|  | time_arrival^1^ | arrivaltime | arrivaltime | admin_arr_tm |
|  | time_inital^1^ | inittime |  |  |
|  | time_treatment^1^ | trettime | fcatime |  |
|  | time_conclusion^1^ | concltime | cottime |  |
|  | time_depature^1^ | deptime | dattime | admin_end_dt |
| diagnosis  classification | diag_code_01-diag_code_12^1^ | diag2_01-diag2_12 | diagnosis1code -diagnosis3code | diag_cd_1-diag_cd_6 |
|  | presentation_freetext^1^ |  | presentingcomplainttext | presenting_complaint |
|  | type_injury^1^ |  | natureofinjurycode |  |
|  | anatomical_area_01- anatomical_area_12^1^ | diaga_01-diaga_12 | bodilylocationofinjurycode | anat_area_cd_1-anat_area_cd_6 |
|  | anatomical_side_01-anatomical_side_12^1^ | diags_01-diags_12 |  | side_cd_1-side_cd_6 |
| treatment  procedure  investigation | investigation_01-investigation_12^1^ | invest2_01-invest2_12 | investigationtype1code - investigationtype3code | invest_cd_1-invest_cd_6 |
|  | treatment_01-treatment_12^1^ | treat2_01-treat2_12 | procedure1code - procedure3code | treat_cd_1-treat_cd_6 |
| costing  resources | dominant_procedure^1^ | domproc | hbtreatmentcode |  |
|  | hrg_nhs^1^ | hrgnhs | hbtreatmentcode |  |
|  | hrg_nhs_svn^1^ | hrgnhsvn |  |  |
| care provider | provider_type^1^ | protype |  | site_cd_of_treat |
|  | provider_code^1^ | procode |  | prov_site_cd |
|  | department_type^1^ | aedepttype |  | triage_cat |

^1^Variable retained without coding scheme. ^2^Variabe retained with a coding scheme.

**Outpatient Services**

| **Category** | **Common Variable Name** | **England – Variable** | **Scotland – Variable** | **Wales – Variable** |
| --- | --- | --- | --- | --- |
| admittance  discharge | appt_date^1^ | apptdate | clinic_date | attend_dt |
|  | attend_type^2^ | atentype | referral_type |  |
|  | attended^2^ | attended | attendance_status | attend_cd |
|  | first_attend^2^ | firstatt | attendance_follow_up | first_attend_cd |
|  | dna_date^1^ | dnadate |  | last_dna_cancel_dt |
|  | appt_priority^2^ | priority |  | priority_type_cd |
|  | req_date^1^ | reqdate | date_referral_received | clinical_ref_dt |
|  | referral_source^2^ | refsourc | referral_source | source_of_ref_cd |
| diagnosis  classification | diag_01-diag_12^1^ | diag_01-diag_12 | main_condition other_condition_1-other_condition_5 | clinical_purpose_cd |
|  | outcome^1^ | outcome |  | outcome_cd |
| treatment  procedure  investigation | operation_01- operation_12^1^ | opertn_01-opertn_12 | main_operation  other_operation_1-other_operation_5 | oper_cd_123 |
| care speciality | staff_type^2^ | stafftyp |  | med_staff_type_cd |
|  | main_speciality^2^ | mainspef | specialty | con_spec_main_cd |
|  | treat_speciality^2^ | tretspef |  | con_spec_cd_of_treat |
| costing  resources | hrg_nhs^1^ | hrgnhs |  |  |
|  | sus_hrg^1^ | sushrg |  |  |
|  | purchase_val^1^ | purval |  |  |
| care provider | pur_code^1^ | purcode |  |  |
|  | provider_code^1^ | procode | gpprac_hb | prov_site_cd |
|  | provider_type^1^ | protype |  | site_cd_of_treat |

^1^Variable retained without coding scheme. ^2^Variabe retained with a coding scheme.
